# Exploring the Potential Relationship Between Global Greenness and DALY Loss Due to Depressive Disorders

**DOI:** 10.3389/fpsyt.2022.919892

**Published:** 2022-06-28

**Authors:** Aji Kusumaning Asri, Hui-Ju Tsai, Wen-Chi Pan, Yue Leon Guo, Chia-Pin Yu, Chi-Shin Wu, Huey-Jen Su, Shih-Chun Candice Lung, Chih-Da Wu, John D. Spengler

**Affiliations:** ^1^Department of Geomatics, National Cheng Kung University, Tainan, Taiwan; ^2^Institute of Population Health Sciences, National Health Research Institutes, Miaoli, Taiwan; ^3^Institute of Environmental and Occupational Health Sciences, National Yang Ming Chiao Tung University, Taipei, Taiwan; ^4^Department of Environmental and Occupational Medicine, National Taiwan University (NTU) and NTU Hospital, Taipei, Taiwan; ^5^National Institute of Environmental Health Sciences, National Health Research Institutes, Miaoli, Taiwan; ^6^School of Forestry and Resource Conservation, National Taiwan University, Taipei, Taiwan; ^7^Department of Psychiatry, National Taiwan University Hospital, National Taiwan University, Taipei, Taiwan; ^8^Department of Environmental and Occupational Health, National Cheng Kung University, Tainan, Taiwan; ^9^Research Center for Environmental Changes, Academia Sinica, Taipei, Taiwan; ^10^Department of Atmospheric Sciences, National Taiwan University, Taipei, Taiwan; ^11^Institute of Environmental Health, School of Public Health, National Taiwan University, Taipei, Taiwan; ^12^Department of Environmental Health, Harvard T.H. Chan School of Public Health, Boston, MA, United States

**Keywords:** country-level, DALY loss, depressive disorders, global analysis, greenness

## Abstract

**Objective:**

Prior studies have shown that greenness can reduce the burden of depressive disorders. However, most were focused on local-scale analyses while limited evaluated globally. We aimed to investigate the association between greenness and the burden of depressive disorders using data from 183 countries worldwide.

**Methods:**

We used the normalized difference vegetation index (NDVI) to estimate greenness. Country-level disability-adjusted life year (DALY) loss due to depressive disorders was used to represent depressive disorder burdens. A generalized linear mixed model was applied to assess the relationship between greenness and depressive disorders after controlling for covariates. Stratified analyses were conducted to determine the effects of greenness across several socio-demographic levels.

**Results:**

The findings showed a significant negative association between greenness and the health burden of depressive disorders with a coefficient of −0.196 (95% CI: −0.356, −0.035) in the DALY changes per interquartile unit increment of NDVI. The stratified analyses suggested beneficial effects of greenness on depressive disorders across sex, various age groups especially for those aged <49 years, with low-income and/or those living in highly urbanized countries.

**Conclusions:**

Our study noted that greenness exposure was significant negative association with the burden of depressive disorders. The findings should be viewed as recommendations for relevant authorities in supporting environmental greenness enhancement to reduce the mental burdens.

## Introduction

Mental illness is one of the most widespread and devastating global problems (1). The impact of mental illness is multiplied because of its ability to trigger other health burdens, such as chronic diseases (2). Even worse, mental illness on its own and in combination with other chronic health conditions is often considered a contributing factor to suicide (3). One of the most common and debilitating mental health burdens, on society is depressive disorders. According to the latest report from World Health Organization (WHO), at a global level, over 264 million people suffer from depressive disorders (4). According to the Global Burden of Disease (GBD) study in 2015, depressive disorders are the second leading causes of global age-specific disability-adjusted life years (DALY) for people aged 20–24 years with the change rate of 0.08 to 0.15 (%), the third for people aged 15–19 years with the change rate of 0.01–0.08 (%), and the fourth also fifth leading cause for people aged 25–44 years (5).

Over time, related issues in the economy, education, and wealth have made socioeconomic status an important focus leading to individual mental health problems, such as depression (6). Previous studies also have documented that the elevated extent of urbanization worldwide is one of the risk factors associated with mental illness. It is estimated that around 60% of the global population will live in cities by 2030 (7). This increasing urbanization will create cities that are more densely populated, further increasing the scarcity of the natural environment in these locations, and subsequently increasing the risk for mental burdens such as depression. The main scarcity of natural environments that often occurs is the limited amount of green space. Previous studies showed that limited green space can increase the concentration of air pollution, which in turn leaves populations more vulnerable to mental illness (8, 9). Numerous studies also reported how green environments could help reduce the burden of depressive disorders (10, 11). In relation to epidemiological studies, estimation of exposure to greenness by using remote sensing data such as normalized difference vegetation index (NDVI) is widely used (12–14). For example, by using multi-temporal NDVI data from MODIS, a study in Korea showed that exposure to greenness could alleviate depressive symptoms in in the seven major cities in Korea (15). Roe et al. also confirmed that exposure to greenness is associated with reductions in stress (16). Residential proximity to green space is often linked with reduced levels of disease burden from both physical and psychological conditions, such as diabetes, stroke, cardiovascular disease, and stress (17, 18). By considering that time spent in green space could possibly affect neural mechanisms, previous studies have also investigated the potential pathways by which exposure to greenness benefits mental health and vitality related to cognitive function (19, 20), as well as the extent to which differences in age and sex can influence these outcomes (21). Furthermore, the psycho-evolutionary theory or stress reduction theory, indicating frequent nature contact could evoke positive stimuli, which in turn promotes a reduction in physiological activation and blocks negative thoughts (22).

Although previous studies have investigated the positive effect of greenness on depressive disorders such as major depressive disorder and dysthymia, most were only focused on local-scale analyses while limited available studies have evaluated the benefits of greenness globally. Knowing that depressive disorders is a pioneer for global psychological burden, in this ecological study, we proposed a novel concept to investigate the association between greenness and the burden of depressive disorders in 183 countries worldwide. The main aimed was to respond to scientific gaps related to the relationship between green exposure and mental health burden, which is mostly only carried out in developed countries. By evaluating the association using multinational data, this study proposed to cover the shortfall in evaluating the relationship between greenness exposure and the burden of depression globally, which includes developed and developing countries. We hypothesized that exposure to greenness is inversely associated with the health burden due to depressive disorders globally. We also hypothesized that the beneficial effects of greenness on depressive disorders varies by age, sex, economic status, and urbanization level. Finally, for this study we focused on population-based proxies, therefore the related findings could serve as a baseline for global studies that support environmental development to improve the quality of health and wellbeing.

## Materials and Methods

### Health Burden Due to Depressive Disorders

**Figure 1:**
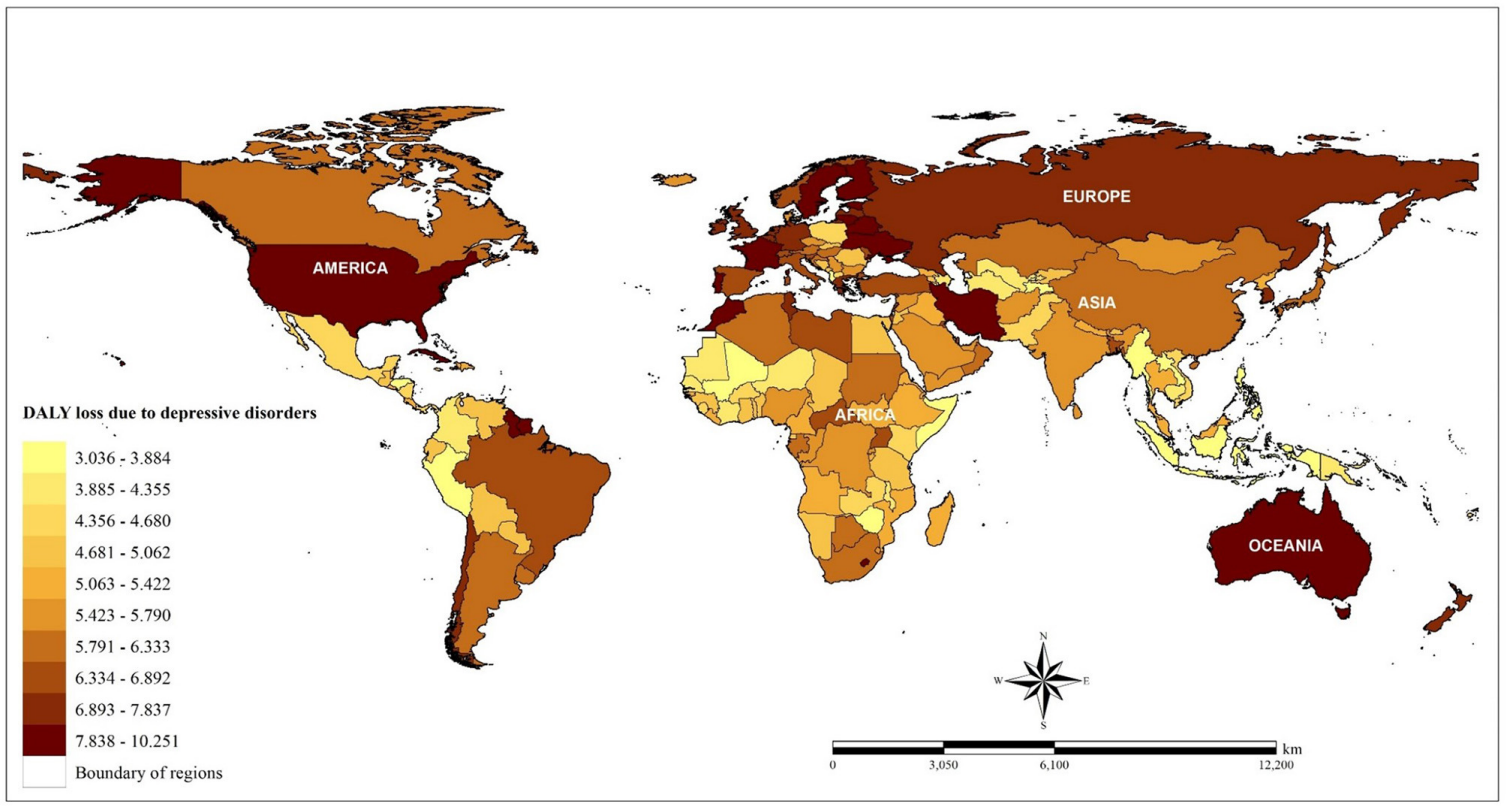
Spatial distribution of DALY loss due to depressive disorders across countries based on the average value from four study periods (2000, 2010, 2015, 2016).

We used the data for disability-adjusted life years (DALY) loss to represent the country-level disease burden due to depressive disorders. This data was provided by the World Health Organization (WHO) from the Global Burden of Disease (GBD) study database. Country-level annual estimation data were available for the four follow-up years (2000, 2010, 2015, and 2016) (www.who.int/healthinfo/global_burden_disease/estimates/en/index1.html). This data is an estimated value and calculated from a combination of medical data, epidemiology, survey, and meta-regression modeling for each country. In brief, DALY is a summary metric of population health burden due to specific disease. It includes two components: years lived with disability (YLD) and years of life lost due to premature mortality (YLL). This data used a new normative standard life table to compute YLL and adjusted for comorbidity to calculate YLD (23). The DALY data is available for the entire population and is grouped by various factors, such as sex and age group (5–14, 15–29, 30–49, 50–59, 60–69, and ≥70 years). According to the database used, we defined depressive disorders using International Classification of Diseases 10th revision codes (F32–F33, F34.1) for non-communicable diseases. In total, 183 WHO member countries across five continents with available data on the health burden of depressive disorders were analyzed. Figure 1 shows the spatial distribution of DALY loss due to depressive disorders. The mental health burden due to depressive disorders was selected as the primary outcome of this study because of the high global burden due to depressive disorders and because previous studies showed that this health burden could trigger an increase in the risk of other health burdens (2, 3).

### Assessment of Greenness

Could cover all regions of the world, the Normalized Difference Vegetation Index (NDVI) measured by a Terra Moderate Resolution Imaging Spectroradiometer (Terra-MODIS) sensor with 1x1 km spatial resolution was used to estimate the presence of greenness in each country (24). The data provided by the National Aeronautics and Space Administration (NASA) includes the monitoring and measuring of vegetation, plants, and biomass production, as well as components of greenness including chlorophyll, canopy structure, and leaf (25). The monthly NDVI used in this study was MOD13A3 version 6 and for a given pixel has a range of values from −1.0 to +1.0. Positive values represent greener vegetation and negative values indicate limit vegetation (26). Since recent studies have indicated an association between negative NDVI values and the proximity to water (27), pixels with negative values were excluded to avoid misclassification bias due to the effects of water. In our analysis, satellite-images with an acquisition date closer to mid-season were collected for January, April, July, and October; the month settings for the data collection take into consideration countries with two and/or four seasons. In total, 292 MODIS NDVI images were used to assess the greenness of the global area covering the 183 selected countries. For image integration, we generated a monthly global greenness map by combining the 292 images. Next, we established similar procedures to assess greenness for the four selected months. Finally, monthly greenness concentrations were calculated to estimate the annual average values of greenness for each country.

**Figure 2:**
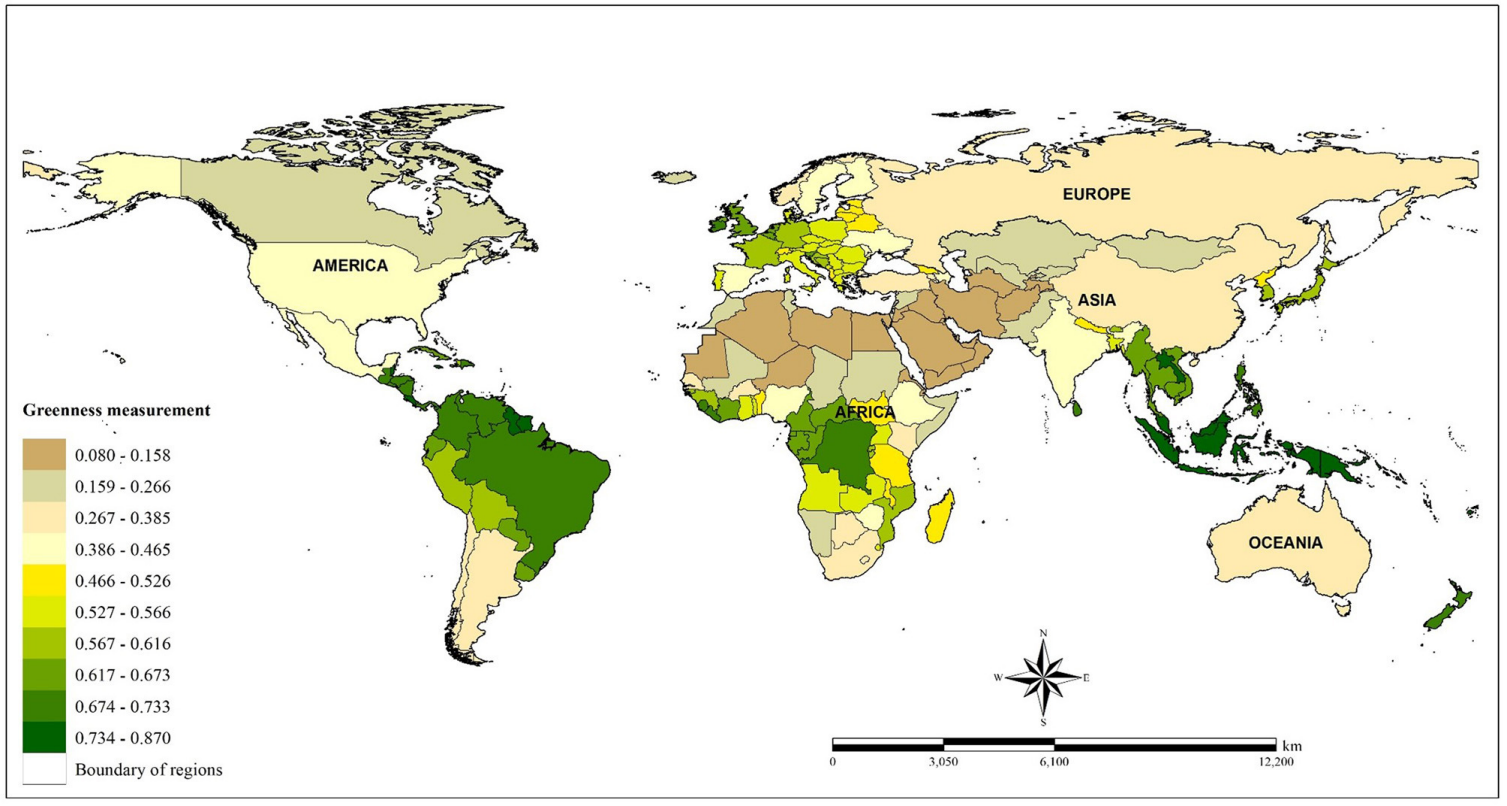
Spatial distribution of global greenness based on average of NDVI (crude) assessment from four study periods (2000, 2010, 2015, 2016).

In all, this process integrated a total of 4,672 images across the four follow-up years (2000, 2010, 2015, and 2016). The spatial distribution of greenness in each country based on the NDVI estimations is shown in Figure 2.

### Potential Risk Factors

Referring to previous literature, several potential variables assessed in each country were considered for model adjustments (Supplementary Table 1). We controlled for demographic covariates including population density, age, and sex provided by United Nations because these factors are often linked with depressive disorders (28, 29). The proportion/ percentage of the educated population on a country-level data obtained from World Bank Group was considered as a protective effect against depressive disorders (30). Because disparity in socioeconomic status is medical health care for each population (31–33), income level data from World Bank Group, as well as the proportion of urban areas in each country, were incorporated into the model. We also considered country-level healthcare expenditure data from World Bank Group (34). Further, sociocultural elements such as religion data provided by United Nations, were also taken into account because they play an important role in helping people adapt to physiological burdens (35). We examined the divorce rate data from United Nations as a social burden since it has been previously linked to depressive disorders (36, 37). Next, we treated blood pressure data from WHO and lifestyle behaviors including smoking and alcohol consumption as covariates obtained from World Bank Group, as suggested by previous studies (38–41). In this study, we also considered exposure to air pollution such as fine particulate matter (PM_2.5_) estimation with spatial resolution 1 × 1 km^2^ provided by The Atmosphere Composition Analysis Group, Dalhousie University (in 2000, 2010, 2015, 2016). This is because recent studies confirmed the negative effects of PM_2.5_ on mental health (42, 43). As a part of covariate adjusted in the association model, country-level PM_2.5_ data is a daily total column of aerosol optical depth retrievals from satellites that was coupled with the GEOS-Chem transport model and geographically weighted regression model (44). Finally, since a prior study confirmed the link between mental disorders and temperature (45), thus, this study also took into account this meteorological factor in the model adjustment.

### Statistical Models and Sensitivity Test

Descriptive statistics were provided to present the country-level characteristics of all covariates examined in this study, including DALY loss of depressive disorders, environmental exposures (greenness and PM_2.5_), demographic factors (population density, sex, and age), economic status, the prevalence rate of education level, urbanization level, the prevalence rates of populations without religion, divorce rate, lifestyle behaviors (alcohol consumption and smoking), blood pressure, healthcare expenditure, and meteorological factors (i.e., temperature).

The main model with adjustment for the above-listed covariates was developed using the Generalized Linear Mixed Model (GLMM) with a penalized quasi-likelihood (PQL) algorithm to determine the relationship between exposure to greenness and the health burden of depressive disorders. The GLMMPQL accounts for both fixed and random effects and provides a flexible approach for analyzing health outcomes (46). In the case where spatial data are available from different areas, GLMMPQL can adjust the overall fixed effects while the structure of correlation is nested within areas, enabling the adjustment of regional differences in e.g., autocorrelation distances, and considering spatial autocorrelation only between observations in the same region (47). Given the values for DALY loss due to depressive disorders clustered in some countries, we added an additional term of “continent” as fixed effect in the GLMMPQL calculation to minimize the biases due to the spatial autocorrelation issue. Then, a Spatial Autocorrelation (Global Moran's I) was performed to examine whether a spatial autocorrelation (SAC) could be detected in the association model (46). By using residual estimation value from the main model, Moran's I Index and both z-score and *p*-value were calculated. An insignificant Moran's I with an index close to 0 and *p* > 0.05 supports the evidence that there is no explicit spatial autocorrelation problem in the developed model. Furthermore, generalized variance-inflation factors (GVIFs) were applied to examine the multicollinearity problem across covariates (48, 49). The GVIFs value was < 4 for all covariates, thus all variables were included in our model adjustment (Supplementary Table 2).

To evaluate the robustness of our main association model, a sensitivity test was applied. We included different covariates in six separate models adjusted by population density, age, sex, and year in order to discern the change in coefficient estimation and significance. Specifically, **Model 1** only includes greenness exposure; **Model 2** includes exposure to greenness and PM_2.5_ exposure; **Model 3** adds education and economic status in addition to exposure to greenness and PM_2.5_ exposure; **Model 4** adds urbanization level and the prevalence rate of the population without religion; **Model 5** adds behavior factors; and **Model 6** considered blood pressure as a risk factor and divorce rates for each country. In this sensitivity analysis, we assumed that there is no significant change in the estimation coefficient or the significance indicator (*p*-value) which indicates the robustness of the main relationship model.

Subsequently, this study conducted stratified analysis based on the level of greenness and socio-demographic factors. First, by using quartile of NDVI (Q1–Q4), the association between greenness in different exposure levels and the burden of depressive disorders were examined. In this case, Q1 represented countries with lower exposure to greenness (as reference), and Q4 represented countries with higher exposure to greenness, quartile 1 (NDVI: 0.085–0.389); quartile 2 (NDVI: 0.390–0.524), quartile 3 (NDVI: 0.525–0.622), and quartile 4 (NDVI: 0.623–0.808). This stratified analysis was considered, given that not all countries have the same green exposure. By completing this analysis, it can be seen whether there is a difference in the effect of the level of green exposure on the outcome. Second, the association between greenness and depressive disorder burdens was examined in different level of socio-demographic factors (50). In this part, we stratified the data by sexes (male and female), six age groups (5–14, 15–29, 30–49, 50–59, 60–69, and ≥70 years), economic statuses (low-income, middle-income, and high-income countries), and urbanization level (low-urban, middle-urban, and high-urban countries).

### Positive-Negative Exposure and Outcome Controls

As a part of validity analysis, positive-negative control variables were used to check the strength of a causal inference of an exposure-outcome association when unobserved factors were thought to be present. Two approaches were used in this study, including a positive-negative outcome control and a positive-negative exposure control. A positive-negative outcome control aimed to identify whether using the same exposure (greenness) and replacing health burden (i.e., DALY loss due to depressive disorders) with other disease burdens from the same dataset could yield consistent results. For positive outcome control, we evaluated the linkage between greenness and the burden of disease due to cardiovascular diseases and for negative outcome control we evaluated Human Immunodeficiency Virus or HIV. Cardiovascular diseases were chosen as a positive outcome control because Yeager's study showed that greenness has a beneficial impact on reducing cardiovascular risk (51). HIV was chosen as a negative outcome control since no studies focused on this issue. In contrast, in the positive-negative exposure control analysis, this study identified whether using studied health burden (i.e., depressive disorders) and replacing greenness exposure could generate a consistent finding. For the positive exposure control, we examined the association between PM_2.5_ exposure and burden of IHD. A prior study confirmed that PM_2.5_ was correlated with an increased risk of depressive disorder burdens (52). Then, wind speed was used for the negative exposure control with the assumption that no association between wind speed and depressive disorder burdens.

All of the spatial and statistical analyses were performed using ArcGIS 10.7.1 (Esri Inc., Redlands, California, United States) and R version 3.6.3 (The R packages Foundation for Statistical Computing, Vienna, Austria). Coefficient estimates with 95% confidence intervals (CI) were reported and *p* < 0.05 were considered statistically significant. Further, to avoid false discovery rate for independent test statistics, adjusted *p*-value (Adj. *p*-value) was then calculated using the more powerful adjustment method proposed by Benjamini and Hochberg (53) and Jafari and Ansari-Pour (54).

## Results

### Descriptive Statistics

Table 1 presents the descriptive statistics of each variable examined in this study. The average global burden due to depressive disorders was 5.62 years [standard deviation (SD): 1.44 years] for the four study periods (2000, 2010, 2015, and 2016). The average amount of greenness—NDVI was 0.49 (SD: 0.21) and estimated per interquartile (IQR = 0.309), the average value was 1.59 (SD: 1.37); average value of PM_2.5_ exposure was 19.45 μg/m^3^ (SD: 16.10 μg/m^3^); the population density of all selected countries was about 163 population per km^2^ (mean ± SD: 163.37 ± 722.47 persons); and almost 50.0% of the global population measured was female.

**Table 1:** Descriptive statistics of variables examined in this study, **(A)** continuous variables and **(B)** categorical variables.

**A**							
**Variable**	**Mean**	**SD**	**Min**	**25th**	**Median**	**75th**	**Max**
DALY loss due to depressive disorders (years)	5.62	1.37	3.04	4.65	5.38	6.47	10.25
**Main exposure**							
Greenness (continuous NDVI)	0.49	0.21	0.08	0.34	0.54	0.65	0.87
Greenness (IQR of NDVI = 0.309)	1.59	0.67	0.25	1.11	1.74	2.11	2.81
**Risk factors**							
Population density (people/km^2^)	163.37	722.84	4.88	19.84	49.87	110.13	1,017.36
Sex (female, %)	49.95	3.05	24.17	49.77	50.32	50.92	54.21
Age 5–14 (yrs, %)	19.17	6.42	8.04	13.22	19.05	25.53	31.28
Age 15–29 (yrs, %)	25.38	4.13	14.52	22.30	26.66	28.17	36.76
Age 30–49 (yrs, %)	25.30	5.26	15.77	21.07	25.94	28.70	52.04
Age 50–59 (yrs, %)	8.62	3.58	2.88	5.30	7.85	12.01	16.08
Age 60–69 (yrs, %)	5.89	3.29	1.14	3.16	4.62	8.59	14.30
Age ≥ 70 (yrs, %)	5.10	3.97	0.39	1.95	3.28	7.75	19.08
Education (%)	83.70	19.68	0.00	72.60	92.80	98.80	100.00
Urbanization level (%)	55.68	22.62	10.84	37.04	55.67	73.71	100.00
No religion (%)	0.04	0.10	0.00	0.00	0.01	0.02	0.77
**Covariates**							
Divorce rate (per 1,000 population)	1.02	1.11	0.00	0.00	0.80	1.72	5.95
Healthcare expenditure (% of GDP)	6.22	2.39	0.00	4.62	6.01	7.84	15.88
Alcohol consumption (liters/population/year)	3.26	3.61	0.00	0.21	2.00	4.82	16.64
Smoking (%)	18.60	13.79	0.00	6.70	19.10	28.30	73.40
Mean systolic blood pressure (mmHg)	126.53	3.39	116.61	124.39	126.65	129.23	134.49
PM_2.5_ (μg/m^3^)	19.45	16.10	0.46	7.34	15.02	27.45	87.53
Temperature (°C)	19.64	8.170	−6.211	11.947	23.276	26.151	29.541
**B**							
**Variable**	**Number (countries)**						**%**
**Economic status**							
Low-Income	80						43.71
Middle-Income	52						28.42
High-Income	51						27.87
**Continent**							
Asia	46						25.14
Africa	54						29.51
America	34						18.58
Europe	39						21.31
Oceania	10						5.46

### Association Models Between Greenness and the Health Burden of Depressive Disorders

A significant negative association between global greenness and depressive disorder burdens were observed (Table 2), with a coefficient of the main model was −0.635 (95% CI: −1.155, −0.115) for the DALY changes per increase in NDVI from 0 to 1 or coefficient estimate was −0.193 (95% CI: −0.356, −0.035) in the DALY changes per interquartile unit increment of NDVI (IQR = 0.309). This finding indicates greenness may have a beneficial effect in dealing with the health burden due to depressive disorders. We consistently found significant negative associations between greenness and health outcome from the first six sensitivity test models.

**Table 2:** Association models between greenness and depressive disorders in the DALY changes per unit increment of NDVI, supported by stratified analysis.

**Model**	Coefficient of NDVI^*^(95% CI)	**Adj. *p*-value**	Coefficient of NDVI^**^(95% CI)	**Adj. *p*-value**
Main Model^a^	−0.635 (−1.155, −0.115)	0.030	−0.196 (−0.356, −0.035)	0.030
**Sensitivity test adjusted for covariates**				
Model 1^b^	−0.633 (−1.101, −0.166)	0.011	−0.196 (−0.340, −0.051)	0.011
Model 2^c^	−0.640 (−1.113, −0.167)	0.013	−0.197 (−0.344, −0.051)	0.013
Model 3^d^	−0.565 (−1.039, −0.091)	0.030	−0.174 (−0.321, −0.028)	0.030
Model 4^e^	−0.562 (−1.036, −0.088)	0.034	−0.173 (−0.320 −0.027)	0.034
Model 5^f^	−0.535 (−1.009, −0.061)	0.039	−0.165 (−0.311, −0.019)	0.039
Model 6^g^	−0.664 (−1.171, −0.158)	0.030	−0.204 (−0.360, −0.047)	0.030

* *Continuous data of NDVI (0–1).*

** *Interquartile or IQR of NDVI (0.309).*

*CI, Confidence interval; NDVI, Normalized different vegetation index.*

a *Control variables included population density, sex (% of females), age, PM*_2.5_, *economic status, the prevalence rate of education, population without religion, the prevalence rate of smoking, alcohol consumption, systolic blood pressure, divorce rate, urbanization level, healthcare expenditure, and temperature.*

b *Adjusted for population density, sex (% of females), and age.*

c *Adjusted for population density, sex (% of females), age, and PM*_2.5_.

d *Adjusted for population density, sex (% of females), age, PM*_2.5_, *economic status, and the prevalence rate of education.*

e *Adjusted for population density, sex (% of females), age, PM*_2.5_, *economic status, the prevalence rate of education, and population without religion.*

f *Adjusted for population density, sex (% of females), age, PM*_2.5_ *exposures, economic status, the prevalence rate of education, population without religion, the prevalence rate of smoking, and alcohol consumption.*

g *Adjusted for population density, sex (% of females), age, PM*_2.5_, *economic status, the prevalence rate of education, population without religion, the prevalence rate of smoking, alcohol consumption, systolic blood pressure, divorce rate, and healthcare expenditure*.

In a detailed assessment, the coefficient estimates for all associated risk factors adjusted in the models were presented in Supplementary Table 3. In the main model for the relationship between greenness and DALY loss due to depressive disorders, several variables other than greenness were identified as significant, such as sex (female) and divorce rate with estimated coefficients (95% CI) were 3.253 and 0.264, respectively. Moreover, after controlled for all risk factors, the findings also suggested that greenness had a significant negative relationship with the health burden due to depressive disorders in countries with the highest exposure to greenness (quartile 4); coefficient estimate was −0.510 (95% CI: −1.017, −0.003), compared to those with the lowest exposure (quartile 1) (Table 3). We also assessed the spatial-autocorrelation effects in the model. As shown in Supplementary Table 4, no statistically significant clustering effects (*p* > 0.05) were found in the developed models.

**Table 3:** Coefficient estimations of greenness by quartile attributed to depressive disorders in multivariable adjusted models.

	**Model 1^a^**		**Model 2^b^**	
	**Coefficient of NDVI (95% CI)**	**Adj. *p*-value**	**Coefficient of NDVI (95% CI)**	**Adj. *p*-value**
Quartile 1 (Q1)^*^ low exposure	Reference	Reference
Quartile 2 (Q2)^*^	−0.201 (−0.690, 0.289)	0.466	−0.410 (−0.878, 0.058)	0.148
Quartile 3 (Q3)^*^	−0.378 (−0.865, 0.109)	0.160	−0.536 (−1.030, −0.042)	0.042
Quartile 4 (Q4)^*^ high exposure	−0.591 (−1.076, −0.106)	0.025	−0.510 (−1.017, −0.003)	0.049

* *Categorical data of NDVI based on the quartile of NDVI.*

*CI, Confidence interval; NDVI, Normalized different vegetation index.*

a *Additional adjustment for population density, sex, age, and PM*_2.5_.

b *Control variables included population density, sex (% of females), age, PM*_2.5_, *Coefficient estimations of greenness by quartile attributed to depressiveeconomic status, the prevalence rate of education, population without religion, the prevalence rate of smoking, alcohol consumption, systolic blood pressure, divorce rate, urbanization level, healthcare expenditure, and temperature*.

### Stratified Analysis by Socio-Demographic Factors

**Figure 3:**
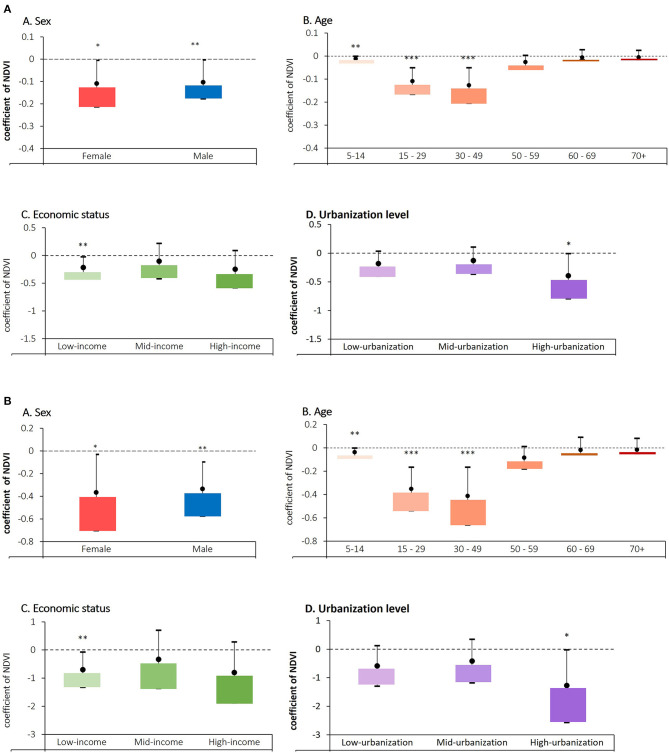
Stratified analysis by sex, age groups, economic status, and urbanization levels in relation to depressive disorders in the DALY changes based on **(A)** crude NDVI; increase in NDVI from 0 to 1, **(B)** interquartile increment unit of NDVI (IQR = 0.309).

After adjusting for potential risk factors, the stratified analyses by sex, age group, economic status, and urbanization level were assessed (Figure 3). We found a significant beneficial effect of greenness on depressive disorders for both females and males with coefficient estimate were −0.109 and −0.104 (95% CI: −0.213, −0.005 and 95% CI: −0.178, −0.003), respectively, in the DALY changes per interquartile unit increment of NDVI. This finding suggesting no sex-based inequality in relation to the impact of greenness on depressive disorder burdens. We found that the burden due to depressive disorders had a negative association with greenness in several age groups, especially the group aged 5–49 years rose to significance. The results from the stratified analysis also showed that significant negative associations of greenness with the health burden of depressive disorders were found in low-income countries and countries with high urbanization levels, coefficient estimate was −0.216 and −0.394 (95% CI), respectively, in the DALY changes per interquartile increment of NDVI.

### Positive-Negative Exposure and Outcome Controls

The results of the associations between positive or negative exposures and depressive disorder burdens were shown in Supplementary Table 5. First, a significant positive association was observed between fine particulate matter (PM_2.5_), a positive exposure control, and depressive disorders. Second, the relationship between the health burden of depressive disorders and wind speed, a negative exposure control, was not significant. We also examined associations between exposure to greenness and positive or negative outcomes. A significant relationship between greenness and cardiovascular diseases, a positive outcome control, was found, whereas a non-significant relationship between greenness and HIV, a negative outcome control, was also observed.

## Discussion

To our knowledge, this is the first global ecological study to investigate the association between greenness and the health burden of depressive disorders across multiple countries. Consistent with our hypotheses, our findings showed a significant negative association between greenness and depressive disorder burdens. In various sensitivity analyses, we consistently obtained similar results and supportive evidence that the higher the greenness exposure, the lower the health burden due to depressive disorders. The significant negative association of exposure to greenness with the burden of depressive disorders remained after accounting for potential country-level risk factors.

Our findings were reinforced by previous local studies. For example, Sarkar et al. observed a protective effect of greenness on depressive disorders, with 4.0% lower odds of major depressive disorder per interquartile increment in NDVI (odds ratio 0.96, 95% CI 0.93–0.99; *p* = 0.004) (50). In America, a study by Bezold et al. also reported that there was a 6% lower incidence rate of high depressive symptoms related to an interquartile range increase in greenness exposure (55). Taking children as the subject, a cohort study in the United States showed that increased exposure to greenness represented by NDVI was associated with a reduced risk of mental disorders such as depression (56). Moreover, the benefit of living in a green environment on mental health related to a lower incidence of depression was also found in South Africa (57). Applying the waitlist-control randomized controlled trial design, a study in Singapore that evaluated the effects of horticultural therapy on Asian adults showed the effectiveness of this therapy in improving mental wellbeing (58). Further, Takayama's study also reported that both people with and without depressive tendencies, forest bathing have a significant positive effect on improving profile of mood states (59). Supportive findings regarding how greenness exposure is associated with a lower risk of depression were also demonstrated by other previous studies (60–68). The beneficial impact of greenness on reduction in the health burden was also identified for physical health diseases such as cardiovascular mortality (51); malignant neoplasms (69, 70); diabetes mellitus (71, 72); respiratory diseases (73, 74); and on the body's metabolic system (75). In general, this study differed from those previous investigations, where the health burden of depressive disorder was represented by the DALY loss metric (considering year loss due to morbidity and mortality) rather than incident or prevalence rate data.

How the presence of greenness could have a significant negative association with the burden of depressive disorders was explained by several plausible reasons. Ulrich (76) emphasized that there are four pieces of evidence indicating the benefits of greenness on health, including (1) a sense of control: green space could provide a sense of control by allowing people a temporary means of physical or mental escape from a stressful conditions; (2) social support: how green space is used to increase social support; (3) physical movement and exercise; and (4) natural positive distractions in mitigating stress, pain, and frightening. In 2008, Ulrich et al. also reported relationships between environmental interventions and health outcomes in two main categories—reduce pain dan reduce stress (77). Furthermore, beneficial pathways of greenness on mental condition confirmed by Kaplan and Kaplan (78) who introduced attention restoration theory (ART). They stated that certain environments including nature are particularly effective at fostering recovery and dealing with fatigue.

We did not find a sex difference in the association between greenness and the burden of depressive disorders. In support of our findings, a systematic review by Bolte et al. showed that no consistent sex differences have been shown related to the impact of greenness on health (79). We did find that the association between greenness and the health burden of depressive disorders extended across various age groups and was significant particularly in those aged 5–49 years. Our findings are supported by several studies suggesting that greenness can reduce the risk of mental health burden among children and adults (80–90), even during pregnancy (91). Contrary to the previous findings on the impact of greenness on the elderly population (13, 92), we did not find significant association effects after age 49 years. Reasonable explanations may be due to changes in socioeconomic roles and decreased mobility to access green spaces at this age, as has also been suggested by Gilroy (93). In the subsequent analyses, we found a significant negative association between exposure to greenness and depressive disorder burdens in low-income and highly urbanized countries, respectively. Similarly, Tomita et al. also proposed the benefits of greenness for mental wellbeing in sub-Saharan Africa which has been experiencing rapid urbanization and economic transition (57). Moreover, Hoffimann's study also confirmed that exposure to greenness has the potential to mitigate health inequalities associated with socioeconomic deprivation (94).

Several strengths were noted in this study. This is the first global ecological study to investigate the association between greenness and the health burden of depressive disorders across 183 countries worldwide. Thus, our findings can serve as a global baseline for future environmental development research studies. Our methodological considerations were sound and included the use of various spatial-statistical approaches to deal with the spatial autocorrelation issue that may be caused by clustering patterns. Moreover, we adjusted our analytical models for several potential confounders with influence on the effectiveness of greenness in minimizing the burden of depressive disorders.

Some limitations also need to be acknowledged. Recognizing that the DALY data provided by WHO is only available at the country-level, not available at the state and/or city levels. A distinct analysis for big countries within-country variability cannot be done. Coming studies could examine the effects of greenness benefits at local communities in the case a finer-resolution DALY data available. In addition, the health burden data represented by DALY obtained from the Global Burden of Disease (GBD) study is multinational and multitemporal data that can be updated. Knowing this issue, considering the latest DALY data is recommended. Although we used greenness data for the same period as the main outcome, due to the limited accessibility of vegetation species data for each country, we did not adjust for the effects of biodiversity in the models. We only used NDVI value to represent total greenness exposure and assumed that all types of greenery had a positive impact on health. In the case of land use or land cover data showing the diversity of greenness is available on a global scale or other green index derived from satellite could estimate spatial greenness exposure, perhaps this could be a better consideration for future studies. Some related factors are unavailable in the global datasets, such as genetic or hereditary disease, health care quality index and ethnicity and/ or race. It will be important to explore the effects of these important factors on depressive disorders in future studies. This study also used an ecological study design, and the measure of exposure was only proxy-based on the average of the whole population. We recognize that the lack of individual data might have some impact on the strength of evidence provided by this study. Finally, the results of this global analysis cannot be directly compared to observational studies and the results should be interpreted with caution.

## Conclusion

Our findings demonstrate a significant negative association between exposure to greenness and the burden of depressive disorders, particularly in low-income and high-urbanized countries. This study is the first to provide evidence for the link between greenness and depressive disorder burdens on a global scale. The findings from this study should serve as a call to policymakers and communities to deploy environmental interventions in terms of helping to deal with the global health burden of depressive disorders, with the potential for positive repercussions for the world.

## Data Availability Statement

The original contributions presented in the study are included in the article/Supplementary Material, further inquiries can be directed to the corresponding author.

## Author Contributions

AKA, S-CCL, H-JS, C-DW, and JDS: conceptualization. AA, H-JT, W-CP, YLG, and C-DW: methodology. AKA and W-CP: formal analysis. AKA, H-JT, and C-DW: writing—original draft preparation. AKA, H-JT, W-CP, YLG, C-PY, C-SW, H-JS, S-CCL, C-DW, and JDS: writing—review and editing. C-DW, S-CCL, and H-JS: supervision and funding acquisition. All authors contributed to the article and approved the submitted version.

## Funding

This study was funded by Academia Sinica, Taiwan, under Trans-disciplinary PM2.5 Exposure Research in Urban Areas for Health-oriented Preventive Strategies (II) (Project No.: AS-SS-110-02).

## Conflict of Interest

The authors declare that the research was conducted in the absence of any commercial or financial relationships that could be construed as a potential conflict of interest.

## Publisher's Note

All claims expressed in this article are solely those of the authors and do not necessarily represent those of their affiliated organizations, or those of the publisher, the editors and the reviewers. Any product that may be evaluated in this article, or claim that may be made by its manufacturer, is not guaranteed or endorsed by the publisher.
