# Supplementary material for: Exploring the Potential Relationship Between Global Greenness and DALY Loss Due to Depressive Disorders
Source: Front Psychiatry. 2022 Jun 28;13:919892. doi: 10.3389/fpsyt.2022.919892 (PMC9273782; doi:10.3389/fpsyt.2022.919892)
Supplement: Supplementary file 1 [file Table_1.DOCX]

**Exploring the Potential Relationship between Greenness Exposure and the Global Burden of Depressive Disorder**

Aji Kusumaning Asri^a^, Hui-Ju Tsai^b^, Wen-Chi Pan^c^, Yue Leon Guo^d,e^, Chia-Pin Yu^f^, Chi-Shin Wu^g^, Huey-Jen Su^h^, Shih-Chun Candice Lung^i,j,k^, Chih-Da Wu^a,e,*^, John D. Spengler^l^

1. Department of Geomatics, National Cheng Kung University, Tainan 70101, Taiwan.
2. Institute of Population Health Sciences, National Health Research Institutes, Miaoli 350, Taiwan.
3. Institute of Environmental and Occupational Health Sciences, National Yang Ming Chiao Tung University, Taipei 11221, Taiwan.
4. Department of Environmental and Occupational Medicine, National Taiwan University (NTU) and NTU Hospital, Taipei, 10055, Taiwan.
5. National Institute of Environmental Health Sciences, National Health Research Institutes, Miaoli 350, Taiwan
6. School of Forestry and Resource Conservation, National Taiwan University, Taipei, 10617, Taiwan.
7. Department of Psychiatry, National Taiwan University Hospital, National Taiwan University, 10055, Taiwan.
8. Department of Environmental and Occupational Health, National Cheng Kung University, Tainan 70101, Taiwan.
9. Research Center for Environmental Changes, Academia Sinica, Taipei, 11529, Taiwan.
10. Department of Atmospheric Sciences, National Taiwan University, Taipei, 10617, Taiwan.
11. Institute of Environmental Health, School of Public Health, National Taiwan University, Taipei, 10055, Taiwan.
12. Department of Environmental Health, Harvard T.H. Chan School of Public Health, Boston, 02115, USA.

* Correspondence to Professor Chih-Da Wu,

No. 1 University Road, Tainan 70101, Taiwan

Telephone: (886) 6-275-7575 ext 63841. Fax: (886) 6-237-5764.

E-mail: [chidawu@mail.ncku.edu.tw](mailto:chidawu@mail.ncku.edu.tw)

**Supplemental Materials**

**Table of contents**

**Table S1.** Data source of potential risk factors.

**Table S2.** The results of generalized variance-inflation factors (GVIFs).

**Table S3.** Complete estimation of the main models developed.

**Table S4.** Significance of spatial autocorrelation tests.

**Table S5.** Positive-negative exposures and outcomes controls.

**Table S1.** Data source of potential risk factors.

| **Variables** | **Description** | **Data Source** | **Year of data** |
| --- | --- | --- | --- |
| Demographic factors (population density, age, gender) | We used country-level demographics data including density of population, age, and sex. In line with the DALY database, we used proportion values from six age groups in the analysis (5-14, 15-29, 30-49, 50-59, 60-69, and >70 years). Sex is represented by percentage of females; females had a higher risk of depression than males (Bebbington, 1996) | United Nations, Department of Economic and Social Affairs. | 2000 - 2016 |
| Economic status | We applied three levels of economic status, including low-income, middle-income, and high-income countries. Classification of economic status for each country was based on the 2016 Atlas gross national income per capita | World Bank Group | 2000 - 2016 |
| Education | We analyzed the proportion/ percentage of the educated population on a country-level | World Bank Group | 2000 - 2016 |
| Urbanization level | We categorized the urbanization level into three groups: low-urbanization, middle-urbanization, and high-urbanization. Level was determined based on the prevalence rate of the urban population in each country. | World Bank Group | 2000 - 2016 |
| No religion | We used data on the prevalence rates of populations without religion identified in each country | United Nations Statistics Division | 2000 - 2016 |
| Divorce rate | We used the prevalence of divorce data in each country that has been standardized by populations aged 15+ years old | United Nations, Department of Economic and Social Affairs | 2008 |
| **Variables** | **Description** | **Data Source** | **Year of data** |
| Continent | We established five regional categories by continent including Africa, America, Asia, Europe, and Oceania to represent the different population characteristics. | United Nations Statistics Division | 2016 |
| Alcohol consumption | We used the average number of alcohol consumption in litres per population | World Bank Group | 2016 |
| Smoking | We used the prevalence rate of smoking data at the country-level | World Bank Group | 2016 |
| Blood pressure | We collected the mean systolic blood pressure data at the country-level | World Health Organization | 2000 - 2016 |
| Healthcare expenditure | Total health expenditure including the provision of health services (preventive and curative), family planning activities, nutrition activities, and emergency aid designated for health were taken into account in our adjustment | World Bank Group | 2000 - 2016 |
| Temperature | We obtained high-resolution grids of monthly temperature data and then we calculated annual average temperature. This dataset is produced by the Climatic Research Unit of the University of East Anglia (UEA) | The Climate Change Knowledge Portal | 2000 - 2016 |
| PM_2.5_ | We estimated PM_2.5_ concentration variations on a global scale with spatial resolution 1x1 km^2^. PM_2.5_ data is a daily total column of aerosol optical depth retrievals from satellites that was coupled with the GEOS-Chem transport model and geographically weighted regression model (van Donkelaar, et al., 2016) | The Atmosphere Composition Analysis Group established by Prof. Randall Martin from Dalhousie University. | 2000 - 2016 |

**Table S2.** The result of generalized variance-inflation factors (GVIFs), multicollinearity test

| **Variables** | **GVIFs** |
| --- | --- |
| **Main exposure** | - |
| Greenness (NDVI) | 1.259 |
| **Covariates** | - |
| Population density (per km^2^) | 1.055 |
| Sex (female %) | 1.363 |
| Age 5 - 14 (yrs, %) | 3.144 |
| Age 15 - 29 (yrs, %) | 1.974 |
| Age 30 - 49 (yrs, %) | 2.219 |
| Age 50 - 59 (yrs, %) | 2.339 |
| Age 60 - 69 (yrs, %) | 2.125 |
| Age > 70 (yrs, %) | 2.330 |
| Economic status | 1.466 |
| Education (%) | 1.374 |
| Urbanization level (%) | 1.235 |
| No religion (%) | 1.024 |
| Divorce rate (%) | 1.207 |
| Healthcare expenditure (% of GDP) | 1.157 |
| Continent | 1.222 |
| Alcohol consumption (liters/population/year) | 1.158 |
| Smoking (%) | 1.154 |
| Mean systolic blood pressure (mmHg) | 1.141 |
| PM_2.5_ (µg/m^3^) | 1.333 |
| Temperature | 1.395 |

**Table S3.** Complete estimation of the main models developed.

| **Variables** | **Coefficient estimates ^a^**  (95% CI) | **Coefficient estimates ^b^**  (95% CI) | |
| --- | --- | --- | --- |
| **Greenness (NDVI)** | **-0.635**  **(-1.155, -0.115)** | **-0.196**  **(-0.356, -0.035)** | |
| PM_2.5_ | 0.001  (-0.003, 0.003) | 0.001  (-0.003, 0.003) | |
| Population density | 0.000  (-0.000, 0.000) | 0.000  (-0.000, 0.000) | |
| Sex (female) | 3.253 (0.528, 5.977) * | 3.253 (0.528, 5.977) * | |
| Age 5 - 14 | 0.050  (0.022, 0.077) ** | 0.050  (0.022, 0.077) ** | |
| Age 15 - 29 | 0.069  (0.046, 0.091) ** | 0.069  (0.046, 0.091) ** | |
| Age 30 - 49 | 0.103  (0.080, 0.126) ** | 0.103  (0.080, 0.126) ** | |
| Age 50 - 59 | 0.097  (0.067, 0.126) ** | 0.097  (0.067, 0.126) ** | |
| Age 60 - 69 | 0.076 (0.048, 0.104) ** | 0.076 (0.048, 0.104) ** | |
| Age ≥ 70 | 0.067  (0.031, 0.103) ** | 0.067  (0.031, 0.103) ** | |
| Alcohol consumption | 0.008 (-0.001, 0.010) | 0.008 (-0.001, 0.010) | |
| Smoking | 0.005 (-0.001, 0.010) | 0.005 (-0.001, 0.010) | |
| Healthcare expenditure | -0.009 (-0.023, 0.005) | -0.009 (-0.023, 0.005) | |
| Education | -0.003 (-0.017, 0.011) | -0.003 (-0.017, 0.011) | |
| **Variables** | **Coefficient estimates ^a^**  (95% CI) | **Coefficient estimates ^b^**  (95% CI) | |
| *Economic status* |  |  | |
| Low-income | reference | |  |
| Middle-income | -0.004 (-0.523, 0.681) | -0.004 (-0.523, 0.681) | |
| High-income | -0.608 (-0.100, 1.502) | -0.608 (-0.100, 1.502) | |
| No religion | 0.002  (-0.003, 0.002) | 0.002  (-0.003, 0.002) | |
| Divorce rate | 0.264 (0.073, 0.455) ** | 0.264 (0.073, 0.455) ** | |
| Urbanization level | 0.001 (-0.003, 0.005) | 0.001 (-0.003, 0.005) | |
| Mean systolic blood pressure | -0.004 (-0.014, 0.021) | -0.004 (-0.014, 0.021) | |
| Temperature | 0.014 (-0.005, 0.034) | 0.014 (-0.005, 0.034) | |

* p-value = <0.05; ** p-value = <0.01; *** p-value = <0.001

CI= Confidence Interval

1. Using continuous data of NDVI (0 to 1).
2. Using interquartile or IQR of NDVI (0.309).

**Table S4.** Significance of spatial autocorrelation tests.

| **Year** | **Moran's Index** | **z-score** | **p-value** |
| --- | --- | --- | --- |
| ***All study periods*** | ***0.004*** | ***0.590*** | ***0.476*** |
| 2000 | 0.011 | 1.179 | 0.238 |
| 2010 | 0.003 | 0.616 | 0.538 |
| 2015 | 0.003 | 0.593 | 0.553 |
| 2016 | 0.002 | 0.560 | 0.575 |

**Table S5.** Positive-negative exposures and outcomes controls.

| **Positive – negative exposures control** | | | | |
| --- | --- | --- | --- | --- |
|  | **PM_2.5_ related to depressive disorders** (positive) | | **Wind speed related to depressive disorders** (negative) | |
|  | Coefficient of CO_2_  (95% CI) | p-value | Coefficient of  Wind speed  (95% CI) | p-value |
| **Model 1 ^a^** | 0.004  (0.001, 0.008) | < 0.05 | 0.029  (-0.095, 0.153) | 0.65 |
| **Model 2 ^b^** | 0.005  (0.001, 0.009) | < 0.05 | 0.066  (-0.053, 0.184) | 0.29 |
| **Positive – negative outcomes control** | | | | |
|  | **Greenness* related to CD**  (positive) | | **Greenness* related to HIV**  (negative) | |
|  | Coefficient of NDVI  (95% CI) | p-value | Coefficient of NDVI (95% CI) | p-value |
| **Model 1 ^a^** | -5.948  (-10.070, -1.821) | <0.001 | 0.581  (-10.940, 10.820) | 0.90 |
| **Model 2 ^c^** | -6.071  (-9.520, -2.622) | <0.001 | 3.725  (-8.233, 15.680) | 0.55 |

CD = Cardiovascular diseases; CI = Confidence Interval; HIV = Human Immunodeficiency Virus.

* using IQR of NDVI (0.309)

1. Additionally, adjusted for population density, sex (% of females), age, and continent.
2. Control variables included population density, sex (% of females), age, PM_2.5_, economic status, the prevalence rate of education, population without religion, the prevalence rate of smoking, alcohol consumption, systolic blood pressure, divorce rate, urbanization level, healthcare expenditure, and temperature.
3. Control variables included population density, sex (% of females), age, economic status (level of income), the prevalence rate of education, the prevalence rate of smoking, alcohol consumption, urbanization rates, systolic blood pressure, and healthcare expenditure.

**References**

Bebbington P. 1996. The origins of sex differences in depressive disorder: Bridging the gap. International Review of Psychiatry. 8(4), 295–332.
https://doi.org/10.3109/09540269609051547

van Donkelaar, A., Martin, R. V, Brauer, M., Hsu, N. C., Kahn, R. A., Levy, R. C., Lyapustin, A., Sayer, A. M., & Winker, D. M. 2016. Global Estimates of Fine Particulate Matter using a Combined Geophysical-Statistical Method with Information from Satellites, Models, and Monitors. Environmental Science & Technology, 50(7), 3762–3772.
https://doi.org/10.1021/acs.est.5b05833.
